# Strong Anionic Repulsion for Fast Na Kinetics in P2‐Type Layered Oxides

**DOI:** 10.1002/advs.202206367

**Published:** 2023-02-07

**Authors:** Dohyeong Kwon, Sung‐Joon Park, Jaewoon Lee, Sangeon Park, Seung‐Ho Yu, Duho Kim

**Affiliations:** ^1^ Department of Mechanical Engineering (Integrated Engineering Program) Kyung Hee University 1732, Deogyeong‐daero, Giheung‐gu, Yongin‐si Gyeonggi‐do 17104 Republic of Korea; ^2^ Department of Chemical and Biological Engineering Korea University 145 Anam‐ro, Seongbuk‐gu Seoul 02841 Republic of Korea

**Keywords:** first‐principles calculations, Ni—Mn binary oxides, oxygen redox, sodium‐ion batteries, sodium‐ion kinetics

## Abstract

An intriguing mechanism for enabling fast Na kinetics during oxygen redox (OR) is proposed to produce high‐power‐density cathodes for sodium‐ion batteries (SIBs) based on the P2‐type oxide models, Na_2/3_[Mn_6/9_Ni_3/9_]O_2_ (NMNO) and Na_2/3_[Ti_1/9_Mn_5/9_Ni_3/9_]O_2_ (NTMNO) using the “potential pillar” effect. The critical structural parameter of NTMNO lowers the Na migration barrier in the desodiated state because the electrostatic repulsion of O(2*p*)—O(2*p*) that occurs between transition metal layers is combined with the chemically stiff Ti^4+^(3*d*)—O(2*p*) bond to locally retain the strong repulsion effect. The NTMNO interlayer distance moderately decreases upon charging with oxygen oxidation, whereas that of NMNO decreases at a much faster rate, which can be explained by the dependence of OR activity on the coordination environment. Fundamental electrochemical experiments clearly indicate that the Ti doping of the bare material significantly improves its rate capability during OR, and detailed electrochemical and structural analyses show much faster Na kinetics for NTMNO than for NMNO. A systematic comparison of the two cathode oxides based on experiments and first‐principles calculations establishes the “potential pillar” concept of not only improving the sluggish Na kinetics upon OR reaction but also harnessing the full potential of the anionic redox for high‐power‐density SIBs.

## Graphical abstract

An intriguing mechanism for facilitating fast Na kinetics upon oxygen redox is suggested to achieve high‐power‐density cathodes for sodium‐ion batteries based on P2‐type oxide models, Na_2/3_[Mn_6/9_Ni_3/9_]O_2_ and Na_2/3_[Ti_1/9_Mn_5/9_Ni_3/9_]O_2_, using the “potential pillar” effect.

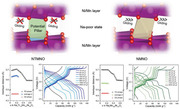

## 1 Introduction

Increasing the energy density of the cathodes in lithium‐ and sodium‐ion batteries (LIBs and SIBs) has been regarded as a crucial task in the development of electric vehicles (EVs) and grid‐scale energy storage systems. Accordingly, a new redox paradigm based on oxygen ions was suggested to overcome the energy limitations of traditional cationic redox reactions utilizing transition metals (Ms) in oxide‐based cathode materials, and its promising electrochemical properties were confirmed by various experiments and computations.^[^ ^1^, ^2^, ^3^, ^4^ ^]^ Based on the fact that the electronic structure of Mn^4+^ ions at an octahedral site coordinated with six oxygen ions is not further oxidizable to Mn^5+^, a representative Li‐excess model, Li_2_MnO_3_ or Li[Li_1/3_Mn_2/3_]O_2_, has been extensively studied to harness the full potential of the oxygen redox (OR) upon charging and discharging.^[^ ^5^, ^6^, ^7^ ^]^ To broaden the possibility of OR reactions, Li_2_MnO_3_‐based Li‐rich layered oxides and even 4*d*‐ and 5*d* cathode models, such as Li_2_RuO_3_ and Li_2_IrO_3_ were investigated.^[^ ^8^, ^9^, ^10^, ^11^, ^12^, ^13^ ^]^ On the other hand, there is a lack of studies on ways to achieve a high power density in OR‐based layered oxides to meet the fast‐charging demands of EVs.^[^ ^14^ ^]^ From the perspective of fast charging, one of the most important factors in increasing the electrochemical rate performance is the interlayer distance (which can also be expressed as the AO_2_ spacing, where A refers to alkali metals such as Li^+^ and Na^+^) between the M layers in layered oxide cathodes.^[^ ^15^ ^]^ Generally, it can be understood that structurally large AO_2_ slabs lead to lower migration barriers for A ions during charge–discharge processes in A‐layered oxides. This intuitive structural point of view revealed the interesting concept that the “cationic repulsion” between M and Li^+^ can be seen as a design parameter in determining the activation barrier of Li ions upon charging and discharging based on the Li[Ni_0.5_Mn_0.5_]O_2_ (LNMO) model.^[^ ^16^ ^]^ In addition, a comparison of IE‐LNMO and SS‐LNMO, where IE and SS refer to ion exchange and solid state, respectively, provided an insight for the design of layered cathodes with high‐rate performance based on the oxygen‐stacking sequence because the former and latter models seemed to be O3‐ and O2‐type layered oxides, respectively. These considerations motivated the advancement of the “cationic repulsion” concept to determine appropriate design strategies based on which species participate in the redox mechanism to attain high‐power‐density OR‐based oxides.

Inspired by the promising OR feature of LIBs, for the first time, an Na[Li_1/3_Mn_2/3_]O_2_ layered oxide with an Mn/Li ratio (R) of 2 was rationally designed to exploit OR reactions and produce high‐energy‐density cathodes for SIBs, and its potential was validated experimentally.^[^ ^17^ ^]^ Like Li‐excess layered oxides for LIBs, the Mn^4+^ ion in the designed Li‐excess Na oxide triggered the OR at ≈4.2 V versus Na^+^/Na upon charging in the energetics of crystal field theory, and various R‐controlled cathodes were investigated.^[^ ^18^, ^19^, ^20^, ^21^, ^22^, ^23^ ^]^ The results of these studies implied that the presence of Li ions in the M layers is an essential condition for the OR of oxide‐based cathodes. However, P2‐type Mn—Ni binary layered oxides were recently re‐examined to unlock the veiled OR during (de)sodiation, and their structures did not contain Li ions in the M layers.^[^ ^24^, ^25^, ^26^, ^27^ ^]^ Although the anionic reaction did not evolve purely, its participation in the charge compensation mechanism was validated by various experimental analyses.^[^ ^28^, ^29^, ^30^ ^]^ In addition, the nonhysteretic oxygen capacities upon cycling were unambiguously observed in Mn—Ni binary oxides, which led to an exciting direction for the practical use of OR.^[^ ^26^, ^28^ ^]^ However, the rate‐performance challenge still remains to harness the full potential of the intriguing OR in Mn—Ni host materials, and its related design approaches have rarely been investigated.

Therefore, this study examined the interesting mechanism denoted as the “potential‐pillar” effect to improve Na kinetics upon an electrochemical OR reaction based on the P2‐type Mn—Ni oxide models, i) Na_1−_ *_x_* [Mn_8/12_Ni_4/12_]O_2_ (NMNO) and ii) Na_1−_ *_x_* [Ti_1/12_Mn_7/12_Ni_4/12_]O_2_ (NTMNO), from a local structure point of view. This concept was systematically understood and confirmed by combining experimental work with first‐principles calculations. The stiff Ti^4+^—O bond as a redox‐inactive component activated the OR derived from the NiO_6_ and MnO_6_ octahedrons in NTMNO and deactivated the anionic reaction from the TiO_6_ octahedron. This mechanism led to the retention of strong O(2*p*)—O(2*p*) repulsion, resulting in a larger interlayer distance than that in NMNO. At a high current density, the charge/discharge capacities during the electrochemical OR for NTMNO were still observed, whereas they disappeared for NMNO. These results were elucidated by detailed electrochemical and structural investigations of the Na kinetics and interlayer distance variations obtained from operando synchrotron X‐ray diffraction (XRD) patterns. Our concrete understanding of the local structure concept provides effective and generalized design strategies for attaining a high power density in OR‐based layered oxides for SIBs and LIBs.

## 2 Results and Discussion

### 2.1 “Potential‐Pillar” Effect in Triggering Fast Na Kinetics upon Oxygen Redox

**Figure 1:**
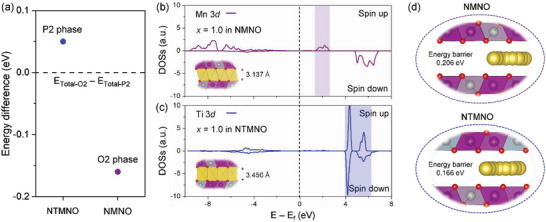
At the fully desoidated state, a) energy differences between O2‐ and P2‐type structures of NTMNO and NMNO, PDOS results of b) Mn(3*d*) in NMNO and c) Ti(3*d*) in NTMNO with calculated interlayer distances, and d) energy barriers and pathways for Na migration in NMNO and NTMNO.

In general, the interlayer distances between MO_2_ layers in layered‐type oxides for LIBs and SIBs have been acknowledged as a paramount factor in determining the A kinetics, directly resulting in the electrochemical rate performance.^[^ ^31^ ^]^ Along this line, the concept denoted as the “cationic repulsion” effect (i.e., the number of M oxidation states is a factor in affecting the electrostatic repulsion between A and M ions during A migration upon charge–discharge processes) was suggested to modulate the A migration barriers in oxide‐based cathodes. In addition, the interlayer distance in P‐type layered oxide is generally larger than that in O‐type layered oxide.^[^ ^32^ ^]^ In light of these, **Figure** **1**a shows that we calculated the energy difference between P2‐ and O2‐type structures at the fully desodiated state (*x* = 1.0) for NMNO and NTMNO, it indicates that the P2—O2 phase transition is thermodynamically favorable for NMNO and not NTMNO (also see Figure S1 for a better understanding, Supporting Information). Based on the phase transition behaviors, the insets in Figure 1b,c shows that we calculated the interlayer distance at the fully desodiated state (*x* = 1.0) in P2‐type Na_1−_ *_x_* [Mn_8/12_Ni_4/12_]O_2_ and Na_1−_ *_x_* [Ti_1/12_Mn_7/12_Ni_4/12_]O_2_ oxide models, and the distance in the Ti‐doped oxide was calculated to be ≈3.450 Å, which was much larger than that in the bare model (≈3.137 Å). It is believed that the Mn ions could be regarded as Mn^4+^ electronic structures at octahedral sites coordinated with six oxygen ions in NMNO, with the Ti ions in the same oxidation state (Ti^4+^) in the Ti‐doped model. To confirm the electronic structures of the cations in the two oxide models, we calculated the partial density of states (PDOSs) of the Mn(3*d*) and Ti(3*d*) electrons at *x* = 1.0 in Na_1−_ *_x_* [Mn_8/12_Ni_4/12_]O_2_ and Na_1−_ *_x_* [Ti_1/12_Mn_7/12_Ni_4/12_]O_2_, as shown in Figure 1b,c. The PDOSs of Mn in the fully desodiated structure show a typical chemical state of Mn^4+^, indicating that the fully oxidized *e*_g_ band is present in the purple region of the conduction band. Consistently, those of Ti with the same charged structure show that of Ti^4+^, revealing that there is no Ti(3*d*) electronic population for a value below the Fermi level, whereas the fully oxidized *e*_g_ and *t*_2g_ bands are observed in the blue region of the conduction band.

Because a large interlayer distance in layered cathodes lowers the migration barrier of A ions during charge–discharge processes,^[^ ^31^, ^32^ ^]^ we calculated the Na‐migration barriers at *x* = 1.0 in Na_1−_ *_x_* [Mn_8/12_Ni_4/12_]O_2_ and Na_1−_ *_x_* [Ti_1/12_Mn_7/12_Ni_4/12_]O_2_, as shown in Figure 1d, and its value in the Ti‐substituted Mn—Ni binary oxide was smaller than that in the pristine material. These results motivated the development of a new concept to elucidate the increase in interlayer distance for NTMNO compared to the reference model (NMNO) because the “cationic repulsion” concept (herein, Mn^4+^—Na^+^ versus Ti^4+^—Na^+^, where the transition metals have the same oxidation state) cannot fully explain the lowered value of the Na‐migration barrier in the NTMNO model. To provide an intriguing mechanism for determining the interlayer distance, the dominant well‐known factor for varying the interlayer distances upon charging was incorporated, indicating the electrostatic repulsion of oxygen ions between the MO_2_ layers. This phenomenon is denoted as the “anionic repulsion” effect. The generalized role of the anion–anion interaction has been attributed to an increase in the interlayer distances upon charging, and this is clearly observed after the initial deintercalation. That is, the “anionic repulsion” effect occurs in vacancy‐sufficient phases in various A‐based layered oxides, where A ions play a physical screening role in suppressing the repulsive force between oxygen layers.^[^ ^33^ ^]^ However, the anionic interaction appears to be insufficient to explain the increased interlayer distance of NTMNO in comparison with that of NMNO, because the charge‐compensation mechanism in the high‐voltage range (>4.0 V vs Na^+^/Na) of Mn—Ni binary oxides for SIBs was recently understood based on the dominant OR accompanied by a partial Ni redox.^[^ ^26^ ^]^ In other words, the OR reaction leads to a decrease in the chemical state of lattice O^2−^ to O*^n^* ^−^ (*n* < 2) upon charging in layered‐type binary oxides.^[^ ^34^ ^]^ Compared to the Mn^4+^(3*d*)—O(2*p*) bond, these considerations suggest that the chemically stiff Ti^4+^(3*d*)—O(2*p*) bond featuring redox‐inactive cationic species upon charging locally retains the strong “anionic repulsion” effect between the M layers in the local structure. This hypothesis is denoted as the “potential‐pillar” effect, which was proposed in our systematic computational work.^[^ ^35^ ^]^ In detail, it conceptually combines the “cationic repulsion” and “anionic repulsion” effects, and this interesting concept can be an exciting design parameter for triggering fast Na kinetics for high‐power‐density in SIBs, especially OR‐related binary Mn—Ni layered oxide cathodes.

### 2.2 Structural Understanding in Association of Redox Inactivity with Interlayer Spacing

**Figure 2:**
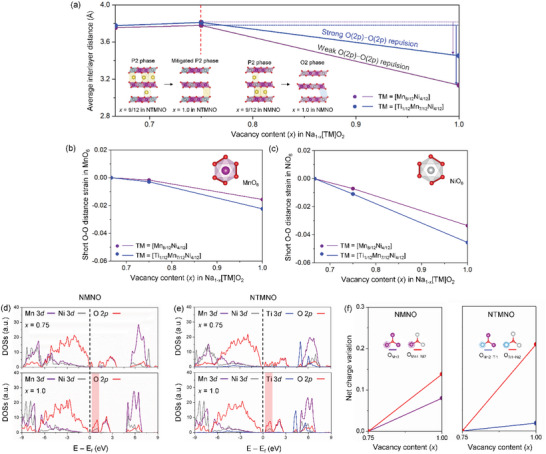
a) Average interlayer distances with varying vacancy contents (*x*) in NTMNO and NMNO from *x* = 0.67 to 1.0, and corresponding atomic models as insets at *x* = 0.75 and 1.0. Strains for short distances in b) MnO_6_ and c) NiO_6_ in the vacancy content for both oxide models. Combined profiles of Mn(3*d*), Ni(3*d*), Ti(3*d*), and O(2*p*) PDOSs at *x* = 0.75 and 1.0 in d) NMNO and e) NTMNO. f) Net charge variations of O coordinated with the transition metals in NMNO and NTMNO during the desodiation.

To structurally understand the “potential‐pillar” effect based on the two oxide models, **Figure** **2**a shows that we investigated the interlayer distances between the MO_2_ layers as a function of the vacancy content (*x*) in Na_1−_ *_x_* [Mn_8/12_Ni_4/12_]O_2_ and Na_1−_ *_x_* [Ti_1/12_Mn_7/12_Ni_4/12_]O_2_ for 0.67 ≤ *x* ≤ 1.0, and the corresponding atomic structures at *x* = 0.75 and 1.0 are illustrated in the insets for a better understanding. Because the P2—O2 phase transition occurs in the former model for a value above *x* = 0.75, the structural parameter diagram shows that there is a slight increase before the breakpoint, whereas a drastic decrease occurs during the P2—O2 phase transition for a vacancy content from *x* = 0.75 to 1.0 in Na_1−_ *_x_* [Mn_8/12_Ni_4/12_]O_2_. Unlike the reference material, the interlayer distance gradually decreases with varying the vacancy content (0.75 ≤ *x* ≤ 1.0) in Na_1−_ *_x_* [Ti_1/12_Mn_7/12_Ni_4/12_]O_2_, which underpins the fact that NTMNO suppresses the formation of the O2‐type structure for a value above *x* = 0.75. In addition, the interlayer distance in NTMNO before *x* = 0.75 is slightly larger than that in NMNO, whereas the difference between the two oxide models is unambiguously enlarged for this vacancy content (0.75 ≤ *x* ≤ 1.0). During the biphasic reaction region showing the dominant OR reaction for the cathode models, the “anionic repulsion” effect is a decisive factor in determining the interlayer distances, which can be understood based on the fact that deeply charged structures exhibiting vacancy‐rich NaO_2_ layers rarely contain Na ions acting in a screening role. Structural observations upon desodiation indicate that the electrostatic repulsion of lattice O(2*p*)—O(2*p*) is stronger for NTMNO than for NMNO. That is, from a local‐structure point of view, the chemically stiff Ti^4+^(3*d*)—O(2*p*) bond suppresses the activation of the OR reaction during the charge compensation mechanism. Therefore, the chemical state of O(2*p*) ions coordinated with Ti^4+^ ions at *x* = 1.0 in Na_1−_ *_x_* [Ti_1/12_Mn_7/12_Ni_4/12_]O_2_ appears to be similar to that at *x* = 0.75 in the oxide, which is not expected to be observed in Na_1−_ *_x_* [Mn_8/12_Ni_4/12_]O_2_. In addition, the strong anionic repulsion induced by the “potential‐pillar” effect mitigates the severe P2—O2 phase transition for this vacancy content (0.75 ≤ *x* ≤ 1.0) in NTMNO, which supports the thermodynamic phase stability of the two oxide models. In contrast, the O(2*p*)—O(2*p*) repulsion weakened by the dominant OR reaction in the reference oxide model causes the rapid collapse of the interlayer spacing, accompanied by the P2—O2 phase transition. Along these lines, we expect an improved rate performance for NTMNO in comparison with that of NMNO, which will be discussed in the experimental sections. From a local structure perspective, the “potential‐pillar” effect can be understood by the O—O distance variation in the MnO_6_ and NiO_6_ octahedrons of Na_1−_ *_x_* [Mn_8/12_Ni_4/12_]O_2_ and Na_1−_ *_x_* [Ti_1/12_Mn_7/12_Ni_4/12_]O_2_, and the corresponding results for this vacancy content (0.67 ≤ *x* ≤ 1.0) are shown in Figure 2b,c. The insets indicate the short O—O distances in the two types of octahedrons. Because an OR above ≈4.0 V versus Na^+^/Na plays a major role in compensating the charge imbalance induced by Na extraction from the host materials, the chemical hardness between M and O was deemed to be a critical factor in effectively mitigating the interlayer distance variation upon the electrochemical OR reaction for NMNO and NTMNO, as discussed in the previous section. The strong chemical bond of Ti—O as a redox‐inactive component gives rise to a larger decrease in the short O—O(MnO_6_) distance in NTMNO than in NMNO over the vacancy range, as shown in Figure 2b. On the other hand, the less hard bond of Mn—O that is derived from the presence of the hybridized Mn^4+^(*t*_2_ *_g_* ^3^)—O(2*p*) band in the low‐energy states in NMNO leads to a decrease in the short O—O distance at a slower rate. This trend was consistently observed in strains with short O—O distances in NiO_6_ with varying vacancy contents in NMNO and NTMNO, as shown in Figure 2c. These results underpin the “potential‐pillar” hypothesis that the chemically stiff Ti—O bond as a redox‐inactive component activates the OR reactions derived from the NiO_6_ and MnO_6_ octahedra in NTMNO and deactivates those from the TiO_6_ octahedra.

To reinforce the intriguing concept induced by the Ti—O chemical bond in Na kinetics, we investigated the electronic structures of the NMNO and NTMNO oxide models. Figure 2d,e shows the combined profiles of Mn, Ni, and the Ti 3*d*‐electron and O 2*p*‐electron at *x* = 0.75 and 1.0 in Na_1−_ *_x_* [Mn_8/12_Ni_4/12_]O_2_ and Na_1−_ *_x_* [Ti_1/12_Mn_7/12_Ni_4/12_]O_2_. The PDOSs at *x* = 1.0 in the pristine material show a clear evolution of the O(2*p*) electronic population in the red‐colored region above the Fermi level, and its intensity is much larger than that of the oxidized Ni(3*d*) electron. This dominant oxygen oxidation has been acknowledged as an unlocked redox mechanism that compensates for the charge imbalance induced by Na extraction for a value above ≈4.0 V versus Na^+^/Na in P2‐type Ni—Mn binary oxides.^[^ ^28^ ^]^ Likewise, the OR trend was observed in the vacancy content for the NTMNO model. For an in‐depth investigation of the OR activities based on the oxide models, oxygen ions were broken down into two types depending on the redox‐(in)active M coordination environment, where M can be Ni, Mn, and Ti in NMNO and NTMNO. The redox‐inactive species were coordinated with Ti and Mn ions, whereas the redox‐active species were coordinated with Ni and Mn ions. In light of these results, we calculated the Bader net charge variation of the two types of oxygen ions in relation to the coordination environments with varying vacancy contents in NMNO and NTMNO, as shown in Figure 2f, where the corresponding atomic configurations are displayed as the insets to provide a better understanding. For the oxygen ion in the redox‐inactive environment, the charge value increased at a gradual rate for NMNO, whereas it rarely increased with the vacancy content for NTMNO. These results implied that the oxygen activity coordinated with Ti and Mn ions was suppressed in the deeply charged state of NTMNO. For the redox‐active environment, the anionic charge variation steeply increased for the Ti‐doped oxide compared to that for the pristine material. The quantitative electronic structure behaviors of the oxygen ions were consistent with the trend of the O—O bond changes during the charging process, which could support the anionic deactivation induced by Ti doping.

### 2.3 Fundamental Understanding of Electrochemical Properties

**Figure 3:**
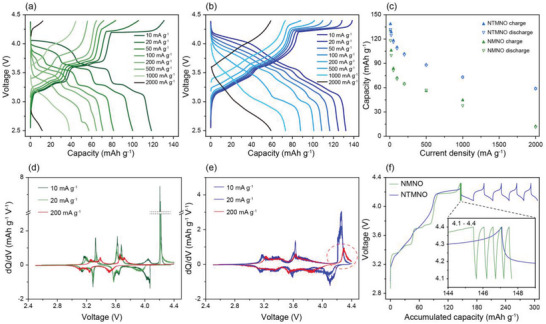
Electrochemical characterization of NMNO and NTMNO cathodes after the first cycle. Voltage profiles in different current densities of a) NMNO and b) NTMNO. c) Comparison of the specific charge capacities and specific discharge capacities of NMNO and NTMNO in different current densities. The differential capacity plots for d) NMNO and e) NTMNO. f) Voltage profiles in the voltage range of 4.1–4.4 V. The inset figure compares the reversibility of the oxygen redox reaction of the cathodes.

Na_2/3_[Mn_6/9_Ni_3/9_]O_2_ (NMNO with *x* = 1/3) and Na_2/3_[Ti_1/9_Mn_5/9_Ni_3/9_]O_2_ (NTMNO with *x* = 1/3) were synthesized via coprecipitation (see the Experimental Section for details). For the material characterization of the synthesized cathodes, powder X‐ray diffraction (XRD) patterns were obtained, and the XRD peaks are well matched with standard Na_0.67_Ni_0.33_Mn_0.67_O_2_ (PDF#54‐0894) patterns with hexagonal in the space group of P6_3_/mmc (Figure S2a, Supporting Information). Moreover, scanning electron microscopy (SEM) images were obtained, as shown in Figure S2b,c (Supporting Information). Both cathodes have angulated platelet‐like shapes with thicknesses of about 400 nm, and their OR activities in the high voltage region were confirmed.^[^ ^36^ ^]^ To compare the prepared cathodes, we investigated their fundamental electrochemical properties under different current densities. As shown in Figure S3 (Supporting Information), NTMNO reveals the reduced charge capacity at the first cycle compared with the NMNO at the current density of 10 mA g^−1^, due to the substitution of electrochemically inactive Ti^4+^ in NMNO. However, followed discharge capacities of NTMNO surpass NMNO's, which could originate from the phase stability and Na^+^/vacancy disordering of NTMNO during (de)sodiation.^[^ ^37^, ^38^, ^39^ ^]^ For further electrochemical characterization based on the rate capabilities, the second charge/discharge voltage profiles of each current density in Figure S3 (Supporting Information) were plotted in **Figure** **3**a,b. Figure 3a presents the galvanostatic charge/discharge profiles of the NMNO electrode at various current densities from 10 to 2000 mA g^−1^. At the lowest current density (10 mA g^−1^), the capacity during the first charging was measured to be 130.90 mAh g^−1^, and that during the subsequent discharge was 118.54 mAh g^−1^. At the highest current density (2000 mA g^−1^), the charging and discharging capacities decreased to 12.22 and 11.63 mAh g^−1^, respectively. In addition, the corresponding plateau of ≈4.20 V at 10 mA g^−1^ upon charging completely disappeared at current densities above 100 mA g^−1^. Compared to the NMNO cathode, the Ti‐doped cathode exhibited a significantly improved performance, as shown in Figure 3b. The charge and discharge capacities of NTMNO at 10 mA g^−1^ were measured to be 138.48 and 132.24 mAh g^−1^, respectively, which were higher than those of NMNO. More importantly, in NTMNO, the charge and discharge capacities at the highest current density were 58.89 and 58.98 mAh g^−1^, respectively, and the OR‐related plateau of ≈4.26 V was still observed at a higher current density of 500 mA g^−1^. These results supported the conclusion that the Ti doping of the bare material not only greatly enhanced its rate capability, but also enabled a reversible OR reaction upon charging and discharging.

To gain an in‐depth understanding of the electrochemical properties, the charge and discharge capacities of the two cathode electrodes are plotted as a function of the current density, as shown in Figure 3c. This graph clearly reveals that Ti incorporation dramatically increases the utilization of the cathode with an increase in the current density. Overall, the degradation of the cathode capacity was mainly derived from the decay in the hybridized OR capacity at high voltages. Nevertheless, it could be inferred that NTMNO was significantly improved kinetically compared to NMNO in the fully charged state. Figure S3 (Supporting Information) presents the stepwise charge/discharge capacities under varying current density conditions as a function of the cycle number for the NMNO and NTMNO electrodes for direct comparison. This diagram indicates that the capacity of NTMNO gradually decreased as the cycle number increased, whereas that of NMNO decreased drastically. In other words, the retention rates of the discharge capacities reached 9.81% and 44.60% for NMNO and NTMNO, respectively. To better understand the OR‐including plateau variation, d*Q*/d*V* analyses of the NMNO and NTMNO electrodes were performed. Figure 3d shows that a clear peak at ≈4.20 V under 10 mA g^−1^ was observed during the charging process for the bare electrode, but there was no peak intensity at 200 mA g^−1^. On the other hand, Figure 3e shows that the Ti‐doped cathode had an unambiguous peak of ≈4.26 V under the same conditions. Intriguingly, at the high current density of 200 mA g^−1^, the corresponding d*Q*/d*V* peak was still observed upon charging the NTMNO. This fundamental understanding of the electrochemical features led to the measurement of the reversibility of sodiation/desodiation in the high‐voltage range for the NMNO and NTMNO electrodes. Figure 3f shows the galvanostatic charge/discharge profiles of the two cathodes as a function of the accumulated capacity in the voltage range (4.1–4.4 V) at 30 mA g^−1^ (0.25 C‐rate) during the initial several cycles. As seen, the Ti‐doped electrode showed outstanding cycle stability and capacity retention, whereas the bare electrode suffered severe degradation under the same conditions.

### 2.4 Electrochemical and Structural Analyses of Na^+^ Kinetics Upon Cycling

**Figure 4:**
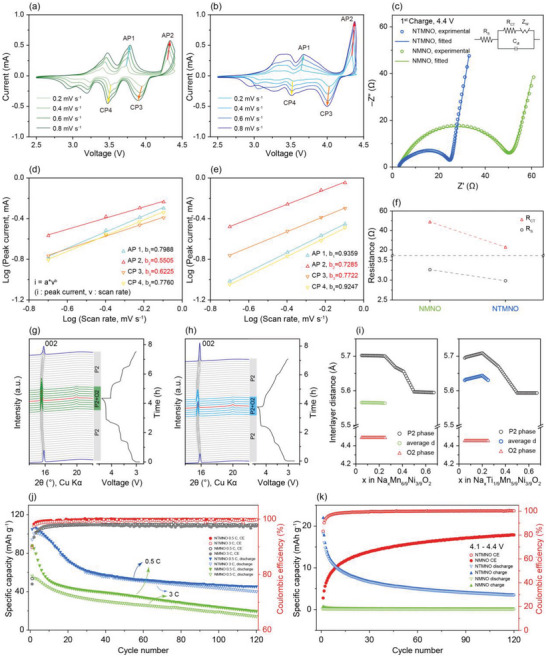
Electrochemical and structural analyses for the understanding of the faster Na^+^ kinetics in NTMNO over NMNO. The CV curves at a different scanning rate of a) NMNO and b) NTMNO. c) EIS analyses and the corresponding equivalent circuit models measured at 1st charge, 4.4 V. f) Resistance from bulk and charge transfer of NMNO and NTMNO. Linear relationships between the peak current and the scanning rate of d) NMNO and e) NTMNO. Operando synchrotron XRD patterns of g) NMNO and h) NTMNO cells under the current density of 30 mA g^−1^. i) Comparison of interlayer distances of the cathodes in terms of P2‐, O2‐ phases and the average interlayer distances. j) Cyclability performances of the cathodes under the C‐rate of 0.5 C and 3 C in the voltage range of 2.5–4.4 V. k) Cyclability performances in the high voltage range of 4.1–4.4 V under the current density of 30 mA g^−1^.

Kinetic analyses based on cyclic voltammetry (CV) were performed to further understand the redox reactions of NMNO and NTMNO. **Figure** **4**a,b displays the CV curves of the cathodes in the voltage window of 2.5–4.4 V with a stepwise scanning rate of 0.2–0.8 mV s^−1^. As the scanning rate increased, the oxidation peaks (anodic peaks, APs) and reduction peaks (cathodic peaks, CPs) also increased. The linear relationship between the measured peak current and scanning rate can be calculated using the following equation^[^ ^40^ ^]^

(1) $$i=a\cdot v^{b}$$

where *b* is the slope of the log(*v*) versus log(*i*) plots. A *b* value of 0.5 indicates that the storage process is diffusion‐controlled. When *b* = 1, the charge storage is a surface‐controlled capacitive process at the surface of the electrode. Figure 4d,e presents the *b* values of NMNO and NTMNO at each redox peak. At ≈3.5 V, where Na^+^/vacancy ordering mainly occurs in Mn—Ni‐based layered oxide cathodes,^[^ ^24^ ^]^ the *b* values of NMNO are *b*_1_ = 0.7988 (AP1) and *b*_4_ = 0.7760 (CP4), while NTMNO exhibits *b*_1_ = 0.9359 and *b*_4_ = 0.9247. These results indicate that the charge–discharge process at a mid‐voltage range value of ≈3.5 V in NTMNO has predominantly surface‐controlled capacitive characteristics. It can be interpreted that Ti incorporation enhanced the kinetics by suppressing the Na^+^/vacancy ordering during cycling. In the high‐voltage range of more than 4.0 V, where the OR reaction mainly occurred, the *b* values were *b*_2_ = 0.5505 (AP2) and *b*_3_ = 0.6225 (CP3) for NMNO, while NTMNO had values of *b*_2_ = 0.7285 and *b*_3_ = 0.7722. These results confirmed that Ti doping also accelerated the oxygen‐redox‐based Na^+^ diffusion mechanism during (de)sodiation. To evaluate the Na^+^ diffusion behaviors of the cathodes during cycling, GITT profiles and Na^+^ diffusion coefficients (*D*_Na+_) are obtained (see Figure S4, Supporting Information). The *D*_Na+_ is determined by altering Fick's 2nd law with some reasonable assumptions. The equation is simplified as follows

(2) $$D=\frac{4}{\pi \tau }{\left(\frac{m_{B}V_{m}}{M_{B}S}\right)}^{2}{\left(\frac{\Delta E_{s}}{\Delta E_{\tau }}\right)}^{2}$$

Here *τ* is the time for an applied galvanostatic current; *m*_B_ and *M*_B_ are the mass and molar weight of the active material. *V*_m_ is the molar volume; *S* is the surface area of the electrode. Δ*E_*τ*_* and Δ*E*_s_ are the equilibrium voltage and total change in the cell voltage *E* during the current pulse, respectively. After a cycle of activation, the GITT protocol is performed at the current density of 30 mA g^−1^, with 15 min of current flux followed by 4 h rest to reach the quasiequilibrium potential. As a result, NTMNO exhibits enhanced *D*_Na+_ during charging. Especially the difference is noticeable over the voltage range of 4.0 V, where the anion redox reaction mainly occurs. The Na^+^ mobility of the cathode is increased by incorporating the Ti‐ion, which coincides with the abovementioned results from CV curves and log(*v*) versus log(*i*) plots.

To compare the charge transfer resistance values, electrochemical impedance spectroscopy (EIS) was conducted for both electrodes at a voltage of 4.4 V in the frequency range of 100 kHz to 100 mHz. Figure 4c shows the EIS spectra of NMNO and NTMNO during the 1st cycle and the corresponding equivalent circuit model. Here, *R*_S_ is the solution (electrolyte) resistance of the cell components, and *R*_CT_ is associated with the charge‐transfer step at the electrode–electrolyte interface. *Z*_W_ is the Warburg impedance originating from the solid‐state Na^+^ diffusion in the electrode, and *C*_dl_ is related to the double‐layer capacitance.^[^ ^41^ ^]^ Figure 4f shows the resistances from the electrolyte (*R*_S_) and charge transfer (*R*_CT_) of NMNO and NTMNO. It is clear that Ti doping induced decreases in both *R*_S_ (from 3.2622 to 2.9840 Ω) and *R*_CT_ (from 48.7891 to 22.8588 Ω), demonstrating that Ti incorporation was beneficial for the Na^+^ kinetics of the cathode. Figure S5 (Supporting Information) displays HR‐TEM images and STEM‐EDS mappings of NMNO and NTMNO. Figure S5a,b (Supporting Information) displays the side view of NMNO and NTMNO with a thickness of ≈400 nm. Additional HADDF‐STEM images of both cathodes along the crystallographic direction of the [001] zone axis are displayed in Figure S5c,d (Supporting Information). The energy‐dispersive EDX maps for NMNO and NTMNO (Figure S5e–h and S5i–m, respectively, Supporting Information) show that the elements are distributed homogeneously.

Figure 4g,h displays operando synchrotron XRD patterns and the corresponding voltage profiles. Both electrodes exhibited the P2—O2 phase transition at a high voltage (≈4.2 V). To make the observation clearer, the phase‐transition regions are colored green and blue for NMNO and NTMNO, respectively. The (002) peaks of NMNO and NTMNO shifted to lower angles during charging, implying that the *c*‐axis expanded as a result of the increased electrostatic repulsion between the adjacent oxygen layers.^[^ ^42^ ^]^ The *c*‐lattice parameters at the pristine state had values of 11.189 and 11.187 Å for NMNO and NTMNO, respectively. During the desodiation, the *c*‐lattice parameters of the P2 phase increased to 11.410 and 11.418 Å for NMNO and NTMNO, respectively. Figure 4i shows the interlayer distances of the cathodes that corresponded to the *d*‐spacing values of the (002) planes. Significantly, the average interlayer distance of NTMNO calculated from the operando synchrotron XRD patterns was larger (5.64 Å) than that of NMNO (5.57 Å), which allowed a wider Na^+^ diffusion pathway during the (de)sodiation and enhanced Na^+^ kinetics and diffusivity during the cycling.^[^ ^43^ ^]^ Figure 4j displays the cycle performances of NTMNO and NMNO at 0.5 and 3 C. In line with the superior rate capability of NTMNO, the cycle fading became relatively low as the C‐rate increased from 0.5 C to 3 C during the cycling. In contrast, NMNO experienced drastic capacity fading under the same conditions. Furthermore, NTMNO exhibited higher coulombic efficiency than NMNO, showing that Ti incorporation also induced better reversibility during the cycling. Figure 4k presents extended cycles at the voltage range of 4.1–4.4 V under the same conditions. It is clear that NTMNO could exploit the oxygen redox reaction in a reversible manner, which occurred in the high voltage region.^[^ ^42^ ^]^ Figure S6 (Supporting Information) displays the electrochemical properties of the cathodes with half‐cell and full‐cell conditions. To prove the practicability of the NTMNO, we conducted full‐cell tests using hard carbon as an anode active material at the current density of 300 mA g^−1^, in the voltage range of 1.5–4.2 V. It is noticeable that NTMNO full cell exhibits good cycling performance and high Coulombic efficiency. The capacity retention of the NTMNO full cell after the 1st formation cycle is 81.1% after 50 cycles when NMNO full cell retained 72.4% at the same conditions. The specific energy density of the NTMNO full cell is about 135 Wh kg^−1^ at 300 mA g^−1^ based on the whole mass of cathode and anode.

## 3 Conclusion

Considering the P2‐type Mn—Ni binary oxide models, an interesting mechanism that combined the anionic repulsion of O(2*p*)—O(2*p*) with the chemical bond of Ti^4+^(3*d*)—O(2*p*) was proposed to enhance the slow kinetics of Na ions upon desodiation via the “potential pillar” effect. First, compared to NMNO, it was confirmed computationally that NTMNO showed a larger interlayer distance, resulting in a lower migration barrier for Na ions in the deeply charged state. Second, the deactivated oxygen ions coordinated with Ti^4+^ ions played a supportive role in retaining the interlayer distance through strong anionic repulsion. Third, charge/discharge oxygen capacities at a high current density unambiguously appeared for the Ti‐doped oxide, but these were not observed for the bare material. Fourth, the CV and EIS experimental results demonstrated that Ti‐incorporation was a significant factor in enhancing the Na kinetics upon the OR, and the operando synchrotron XRD analysis identified a larger interlayer distance for NTMNO than for NMNO. This intriguing concept is expected to be a global strategy for enabling a fast oxygen capacity by modulating the intrinsic local structure of Na‐layered oxides in high‐power‐density cathodes for SIBs and LIBs, and incorporating various redox‐inactive transition metals into OR‐based cathodes will lead to the similar effect on their local structures.

## 4 Experimental Section

### First‐Principles Calculations

The first principles calculation was conducted by using Vienna Ab‐initio Simulation Package (VASP) based on density functional theory (DFT). The generalized gradient approximation (GGA) was applied to all calculations with Perdew–Bruke–Ernzerhof (PBE) functional under spin‐polarized and electronic exchange correlation conditions. The Hubbard U value was considered in 3d transition metals that refer to Mn (4.64), Ni (6.7), and Ti (4.2), respectively. To more accurately describe the Na^+^, the 3s‐ and six 2p‐electrons Na pseudopotential was used. To obtain optimized atomic structures, the calculations were relaxed using a plane‐wave set energy cut‐off 550 eV with the pseudopotentials of the projector augmented wave (PAW). The reciprocal k‐points mesh were set with 4 × 2 × 1 by the Monkhorst–Pack method and converged criterion within 10^−5^ eV and within 10^−2^ eV Å^−1^ in interatomic forces convergence. For an accurate result of electron configuration, the hybrid functional (HF) calculation of Heyd, Scuseria, and Ernzerhof (HSE06) was adopted for correcting the self‐interaction errors between transition metals and oxygen.

### Synthesis of the Cathode Materials

Na_2/3_[Mn_6/9_Ni_3/9_]O_2_ (NMNO) and Na_2/3_[Ti_1/9_Mn_5/9_Ni_3/9_]O_2_ (NTMNO) were synthesized by the coprecipitation method. For the precursor solution, Mn(NO_3_)_2_∙6H_2_O (98%, Aldrich) and Ni(NO_3_)_2_∙6H_2_O (98%, Aldrich) were mixed in deionized water. The molar ratio of the precursor solution was n(Mn):n(Ni) = 2:1 for NMNO and n(Mn):n(Ni) = 5:3 for NTMNO. The mixed solution was dropped into 0.3 mol of NH_4_HCO_3_ solution and then 1 mol L^−1^ Na_2_CO_3_ (98%; Aldrich) was added. The solutions stirred at 600 rpm for 6 h at 90 °C. Then the precipitated particles were centrifuged and washed with DI water and ethanol several times, and then dried in a vacuum oven at 120 °C for 6 h. Obtained transition metal carbonates were mixed with Na_2_CO_3_ (98%; 2 wt% excess; Aldrich). Also TiO_2_ (98%, Aldrich) was added for Ti‐doping. The mixtures were sintered at 550 °C for 5 h then 900 °C for 12 h and finally cooled to room temperature.

### Electrochemical Measurements

CR2032 coin cells were used to evaluate the electrochemical properties of the prepared cathode materials. To fabricate the cathode, a slurry consisting of the active material, super P, and polyvinylidene fluoride (PVDF) in a weight ratio of 8:1:1 was dissolved into N‐methyl‐pyrrolidinone (NMP) and then coated on Al foil by a doctor blade. The loading mass of the active material was about 3 mg cm^−2^. 1.0 m NaClO_4_ in EC:PC = 1:1 with 2 vol% FEC was used as an electrolyte. Sodium metal was used as the counter and reference electrodes and porous glass fiber (Whatman, GF/C) was used as the separator for the half cell tests. The electrochemical tests were conducted on a WonATech cycler workstation at 25 °C. For the full cell test, the hard carbon anode was fabricated by a mixture of hard carbon and PVDF in a weight ratio of 4:1, which was presodiated with Na metal electrochemically. The N/P ratio of the two electrodes was optimized at 1.2. The specific energy density of the full cell was calculated based on the whole mass of the cathode and anode in the voltage range of 1.5–4.2 V at the current density of 300 mA g^−1^, at 25 °C.

### Material Characterizations

The powder XRD patterns were obtained by an X‐ray diffractometer (D‐MAX2500‐PC) in the 2*θ* range of 10°–80° using Cu K*α* radiation (*λ* = 1.5405 Å). The morphology of cathode materials was characterized by scanning electron microscopy (JSM‐7800F Prime). Transmission electron microscopy (TEM) images were obtained by using a field emission transmission electron microscope (FE‐TEM, Tecnai G2 F30 S‐Twin, FEI). Also, High‐angle annular dark‐field scanning transmission electron microscopy (HAADF‐STEM) was conducted with an FEI XFEG‐Titan Themis^3^ Double Cs & Mono. The interlayer distances are obtained via converting *c*‐lattice parameter data measured by operando synchrotron XRD patterns. The measurements were conducted in Pohang Accelerator Laboratory (PAL) on beamline 3D. The obtained 2D MAR345 diffraction images were converted using the Fit2D program based on LaB6 diffraction pattern as a calibration parameter. The operando synchrotron XRD patterns are converted by the Rietveld refinement method using FullProf software. The comparison of the phase transition ratio was based on scale factor data obtained by the software.

## Conflict of Interest

The authors declare no conflict of interest.

## Supporting information

Supporting Information

## Data Availability

Research data are not shared.

## References

[1] Y. Koyama , I. Tanaka , M. Nagao , R. Kanno , J. Power Sources 2009, 189, 798.

[2] M. Sathiya , G. Rousse , K. Ramesha , C. P. Laisa , H. Vezin , M. T. Sougrati , M. L. Doublet , D. Foix , D. Gonbeau , W. Walker , A. S. Prakash , M. Ben Hassine , L. Dupont , J. M. Tarascon , Nat. Mater. 2013, 12, 827. 23852398 10.1038/nmat3699

[3] J. Lee , A. Urban , X. Li , D. Su , G. Hautier , G. Ceder , Science 2014, 343, 519. 24407480 10.1126/science.1246432

[4] D. H. Seo , J. Lee , A. Urban , R. Malik , S. Kang , G. Ceder , Nat. Chem. 2016, 8, 692. 27325096 10.1038/nchem.2524

[5] P. Kalyani , S. Chitra , T. Mohan , S. Gopukumar , J. Power Sources 1999, 80, 103.

[6] S. M. Kang , D. Kim , K.‐S. Lee , M.‐S. Kim , A. Jin , J.‐H. Park , C.‐Y. Ahn , T.‐Y. Jeon , Y. H. Jung , S.‐H. Yu , J. Mun , Y.‐E. Sung , Adv. Sci. 2020, 7, 2001263. 10.1002/advs.202001263 PMC7435253 32832368

[7] K. Kubobuchi , M. Mogi , H. Ikeno , I. Tanaka , H. Imai , T. Mizoguchi , Appl. Phys. Lett. 2014, 104, 053906.

[8] N. Guerrini , L. Jin , J. G. Lozano , K. Luo , A. Sobkowiak , K. Tsuruta , F. Massel , L. C. Duda , M. R. Roberts , P. G. Bruce , Chem. Mater. 2020, 32, 3733.

[9] F. Zheng , S. Zheng , P. Zhang , X. Zhang , S. Wu , Y. Yang , Z. Zhu , J. Phys. Chem. C 2019, 123, 13491.

[10] P. E. Pearce , G. Assat , A. Iadecola , F. Fauth , R. Dedryvère , A. Abakumov , G. Rousse , J.‐M. Tarascon , J. Phys. Chem. C 2020, 124, 2771.

[11] E. McCalla , A. M. Abakumov , M. Saubanère , D. Foix , E. J. Berg , G. Rousse , M. L. Doublet , D. Gonbeau , P. Novák , G. Van Tendeloo , R. Dominko , J. M. Tarascon , Science 2015, 350, 1516. 26680196 10.1126/science.aac8260

[12] P. E. Pearce , A. J. Perez , G. Rousse , M. Saubanère , D. Batuk , D. Foix , E. McCalla , A. M. Abakumov , G. Van Tendeloo , M.‐L. Doublet , J.‐M. Tarascon , Nat. Mater. 2017, 16, 580. 28250444 10.1038/nmat4864

[13] L. Li , F. C. Castro , J. S. Park , H. Li , E. Lee , T. D. Boyko , J. W. Freeland , Z. Yao , T. T. Fister , J. Vinson , E. L. Shirley , C. Wolverton , J. Cabana , V. P. Dravid , M. M. Thackeray , M. K. Y. Chan , Chem. Mater. 2019, 31, 4341.

[14] P. Liu , W. He , Y. Cheng , Q. Wang , C. Zhang , Q. Xie , J. Han , Z. Qiao , H. Zheng , Q. Liu , L. Wang , B. Qu , Q. Luo , Z. Z. Zhu , D. L. Peng , J. Phys. Chem. Lett. 2020, 11, 2322. 32141759 10.1021/acs.jpclett.9b03871

[15] M. Weiss , R. Ruess , J. Kasnatscheew , Y. Levartovsky , N. R. Levy , P. Minnmann , L. Stolz , T. Waldmann , M. Wohlfahrt‐Mehrens , D. Aurbach , M. Winter , Y. Ein‐Eli , J. Janek , Adv. Energy Mater. 2021, 11, 2101126.

[16] K. Kang , Y. S. Meng , J. Bréger , C. P. Grey , G. Ceder , Science 2006, 311, 977. 16484487 10.1126/science.1122152

[17] D. Kim , M. Cho , K. Cho , Adv. Mater. 2017, 33, 1701788. 10.1002/adma.201701788 28635039

[18] K. Zhang , D. Kim , Z. Hu , M. Park , G. Noh , Y. Yang , J. Zhang , V. W. Lau , S.‐L. Chou , M. Cho , S.‐Y. Choi , Y.‐M. Kang , Nat. Commun. 2019, 10, 5023. 30617270 10.1038/s41467-018-07646-4 PMC6323141

[19] K. Du , J. Zhu , G. Hu , H. Gao , Y. Li , J. B. Goodenough , Energy Environ. Sci. 2016, 9, 2575.

[20] S. Koo , I.‐H. Ko , J. Lee , S.‐M. Kang , S.‐H. Yu , D. Kim , ChemElectroChem 2021, 8, 1464.

[21] R. A. House , U. Maitra , L. Jin , J. G. Lozano , J. W. Somerville , N. H. Rees , A. J. Naylor , L. C. Duda , F. Massel , A. V. Chadwick , S. Ramos , D. M. Pickup , D. E. McNally , X. Lu , T. Schmitt , M. R. Roberts , P. G. Bruce , Chem. Mater. 2019, 31, 3293.

[22] X. Rong , E. Hu , Y. Lu , F. Meng , C. Zhao , X. Wang , Q. Zhang , X. Yu , L. Gu , Y. S. Hu , H. Li , X. Huang , X. Q. Yang , C. Delmas , L. Chen , Joule 2019, 3, 503.

[23] G. H. Yoon , S. Koo , S. J. Park , J. Lee , C. Koo , S. H. Song , T. Y. Jeon , H. Kim , J. S. Bae , W. J. Moon , S. P. Cho , D. Kim , S. H. Yu , Adv. Energy Mater. 2022, 12, 2103384.

[24] A. Gutierrez , W. M. Dose , O. Borkiewicz , F. Guo , M. Avdeev , S. Kim , T. T. Fister , Y. Ren , J. Bareño , C. S. Johnson , J. Phys. Chem. C 2018, 122, 23251.

[25] W. Zhao , A. Tanaka , K. Momosaki , S. Yamamoto , F. Zhang , Q. Guo , H. Noguchi , Electrochim. Acta 2015, 170, 171.

[26] K. Dai , J. Mao , Z. Zhuo , Y. Feng , W. Mao , G. Ai , F. Pan , Y. de Chuang , G. Liu , W. Yang , Nano Energy 2020, 74, 104831.

[27] F. Yu , S. Zhang , C. Fang , Y. Liu , S. He , J. Xia , J. Yang , N. Zhang , Ceram. Int. 2017, 43, 9960.

[28] C. Koo , D. Kwon , S. J. Park , J. Lee , G. H. Yoon , S. Hyun Song , T. Y. Jeon , H. Kang , H. Kim , D. Kim , S. H. Yu , Chem. Eng. J. 2022, 446, 137429.

[29] C. Ma , J. Alvarado , J. Xu , R. J. Clément , M. Kodur , W. Tong , C. P. Grey , Y. S. Meng , J. Am. Chem. Soc. 2017, 139, 4835. 28271898 10.1021/jacs.7b00164

[30] H. Yoshida , N. Yabuuchi , K. Kubota , I. Ikeuchi , A. Garsuch , M. Schulz‐Dobrick , S. Komaba , Chem. Commun. 2014, 50, 3677. 10.1039/c3cc49856e 24514951

[31] H. Sun , Z. Cao , T. Wang , R. Lin , Y. Li , X. Liu , L. Zhang , F. Lin , Y. Huang , W. Luo , Mater. Today Energy 2019, 13, 145.

[32] N. Yabuuchi , K. Kubota , M. Dahbi , S. Komaba , Chem. Rev. 2014, 114, 11636. 25390643 10.1021/cr500192f

[33] G. Choi , J. Lee , D. Kim , ACS Appl. Mater. Interfaces 2020, 12, 29203. 32491823 10.1021/acsami.0c04212

[34] B. Mortemard De Boisse , G. Liu , J. Ma , S. I. Nishimura , S. C. Chung , H. Kiuchi , Y. Harada , J. Kikkawa , Y. Kobayashi , M. Okubo , A. Yamada , Nat. Commun. 2016, 7, 11379. 27088834 10.1038/ncomms11397 PMC4837481

[35] J. Lee , S. Park , G. Choi , D. Kwon , J. Kim , H. Kim , M. Cho , D. Kim , Adv. Energy Mater. 2022, 12, 2201319.

[36] S. J. Park , J. Lee , G. H. Yoon , C. Koo , S. H. Lee , S. Koo , D. Kwon , S. H. Song , T. Y. Jeon , H. Baik , H. Kim , D. Kim , S. H. Yu , Energy Storage Mater. 2022, 53, 340.

[37] D. Pahari , S. Puravankara , J. Power Sources 2020, 455, 227957.

[38] P. F. Wang , H. R. Yao , X. Y. Liu , Y. X. Yin , J. N. Zhang , Y. Wen , X. Yu , L. Gu , Y. G. Guo , Sci. Adv. 2018, 4, eaar6018. 29536049 10.1126/sciadv.aar6018 PMC5844706

[39] H. Wang , M. Gu , J. Jiang , C. Lai , X. Ai , J. Power Sources 2016, 327, 653.

[40] Y. Xiao , Y. F. Zhu , H. R. Yao , P. F. Wang , X. D. Zhang , H. Li , X. Yang , L. Gu , Y. C. Li , T. Wang , Y. X. Yin , X. D. Guo , B. H. Zhong , Y. G. Guo , Adv. Energy Mater. 2019, 9, 1803978.

[41] Y. Abe , N. Hori , S. Kumagai , Energies 2019, 12, 4507.

[42] S. J. Park , J. Lee , I. H. Ko , S. Koo , S. H. Song , C. Koo , G. H. Yoon , T. Y. Jeon , H. Kim , D. Kim , S. H. Yu , Energy Storage Mater. 2021, 42, 97.

[43] F. Ding , C. Zhao , D. Xiao , X. Rong , H. Wang , Y. Li , Y. Yang , Y. Lu , Y. S. Hu , J. Am. Chem. Soc. 2022, 144, 8286. 35472274 10.1021/jacs.2c02353

