# Supplementary material for: Strong Anionic Repulsion for Fast Na Kinetics in P2‐Type Layered Oxides
Source: Adv Sci (Weinh). 2023 Feb 7;10(10):2206367. doi: 10.1002/advs.202206367 (PMC10074072; doi:10.1002/advs.202206367)
Supplement: Supplementary file 1 — Supporting Information [file ADVS-10-2206367-s001.pdf]

Supporting Information

Strong anionic repulsion for fast Na kinetics in P2-type layered oxides

Dohyeong Kwon[a,‡], *Sung-Joon Park*[b,‡], *Jaewoon Lee*[a], *Sangeon Park*[a], *Seung-Ho Yu*[b,*] *and Duho Kim*[a,*]

‡ These authors contributed equally to this work

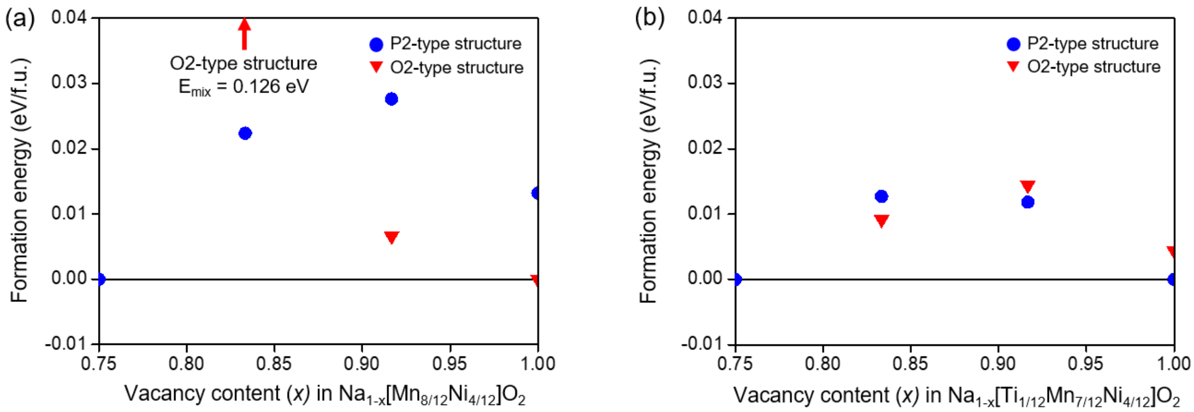

Figure S1. Formation energies of mixing enthalpy as functions of vacancy content (x) from x = 0.75 to x = 1.0 in (a) NMNO and (b) NTNMO, and their values are calculated based on all possible positions of Na ions and their vacancies. In thermodynamic point of view, the energy diagrams indicate that the P2–O2 phase transition occurs for NMNO, whereas it is not favorable for NTMNO.

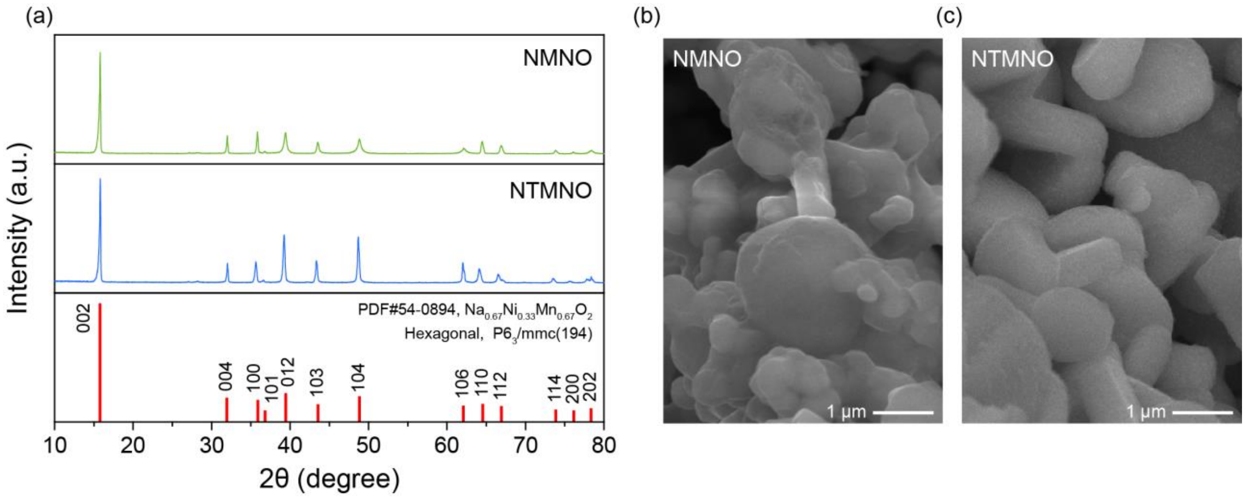

Figure S2. Material characterizations of the cathode active materials. (a) Powder XRD patterns of NMNO and NTMNO. SEM images of as-prepared (b) NMNO and (c) NTMNO.

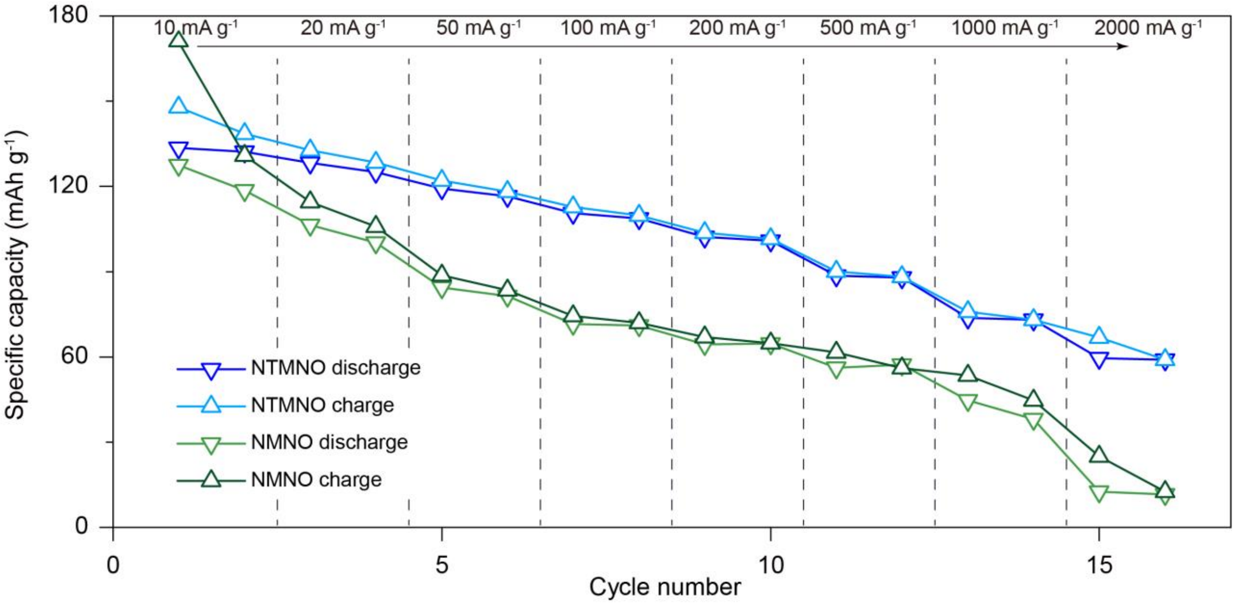

Figure S3. Rate capability data of NMNO and NTMNO with different current densities. 2 cycles of galvanostatic charge/discharge processes were conducted at each current density condition in the voltage range of 2.5 – 4.4 V.

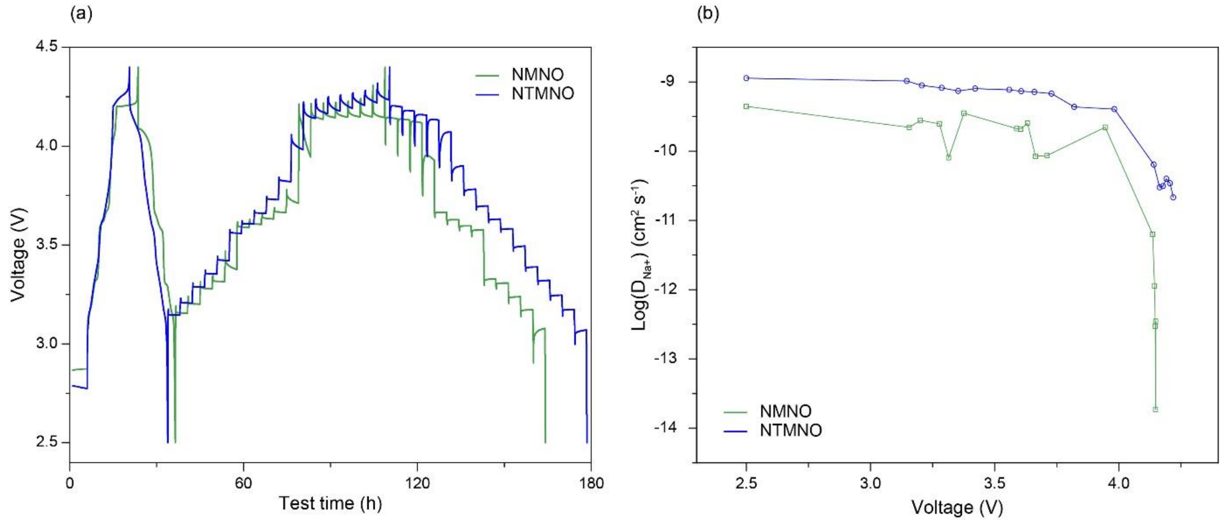

Figure S4. (a) Galvanostatic intermittent titration technique (GITT) curves after a cycle of activation process in the voltage range of 2.5 – 4.4 V. (b) Diffusion coefficient plots of NMNO and NTMNO at charge process, calculated based on the GITT results.

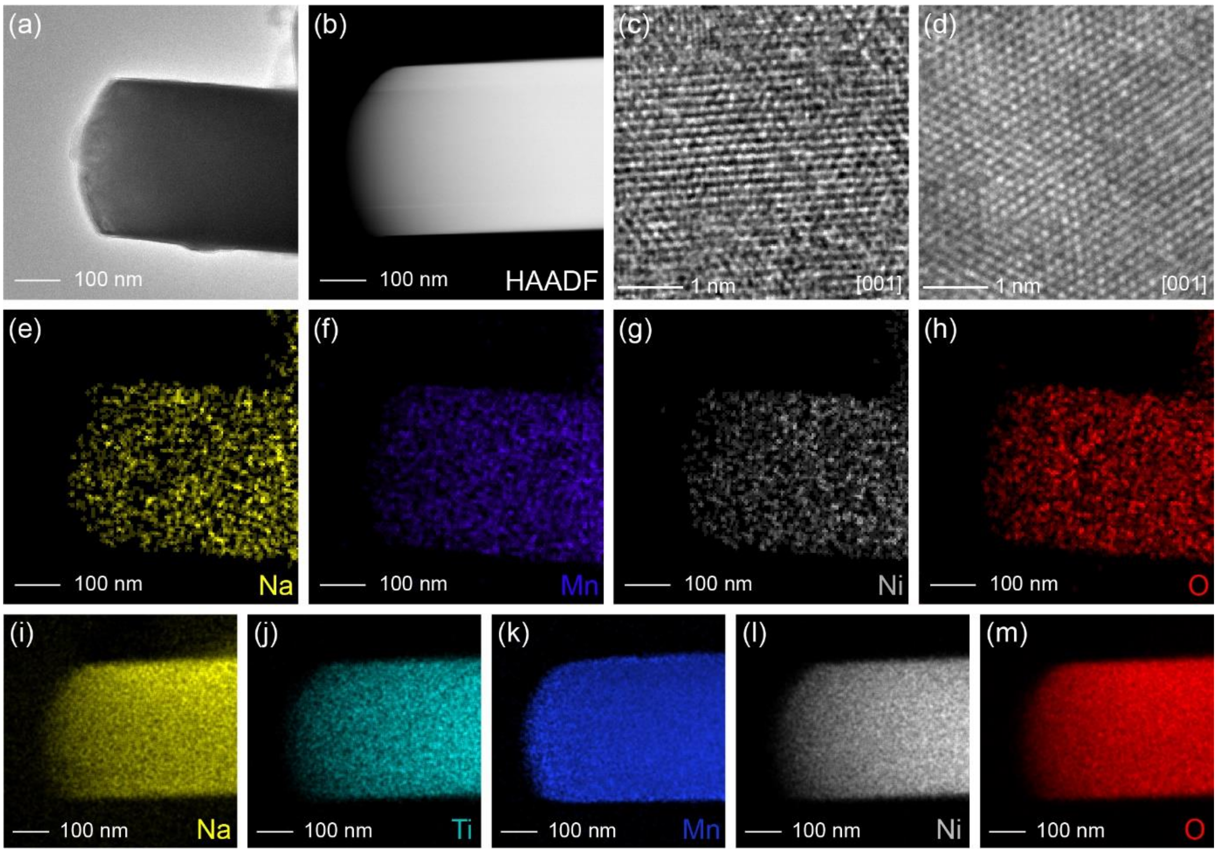

Figure S5. HR-TEM image of (a) NMNO with the side view and HADDF-STEM image of (b) NTMNO. Magnified HADDF-STEM images with [001] projection of (c) NMNO and (d) NTMNO. EDX mappings of NMNO in elements of (e) Na, (f) Mn, (g) Ni, (h) O, and NTMNO in elements of (i) Na, (j) Ti, (k) Mn, (l) Ni, (m) O.

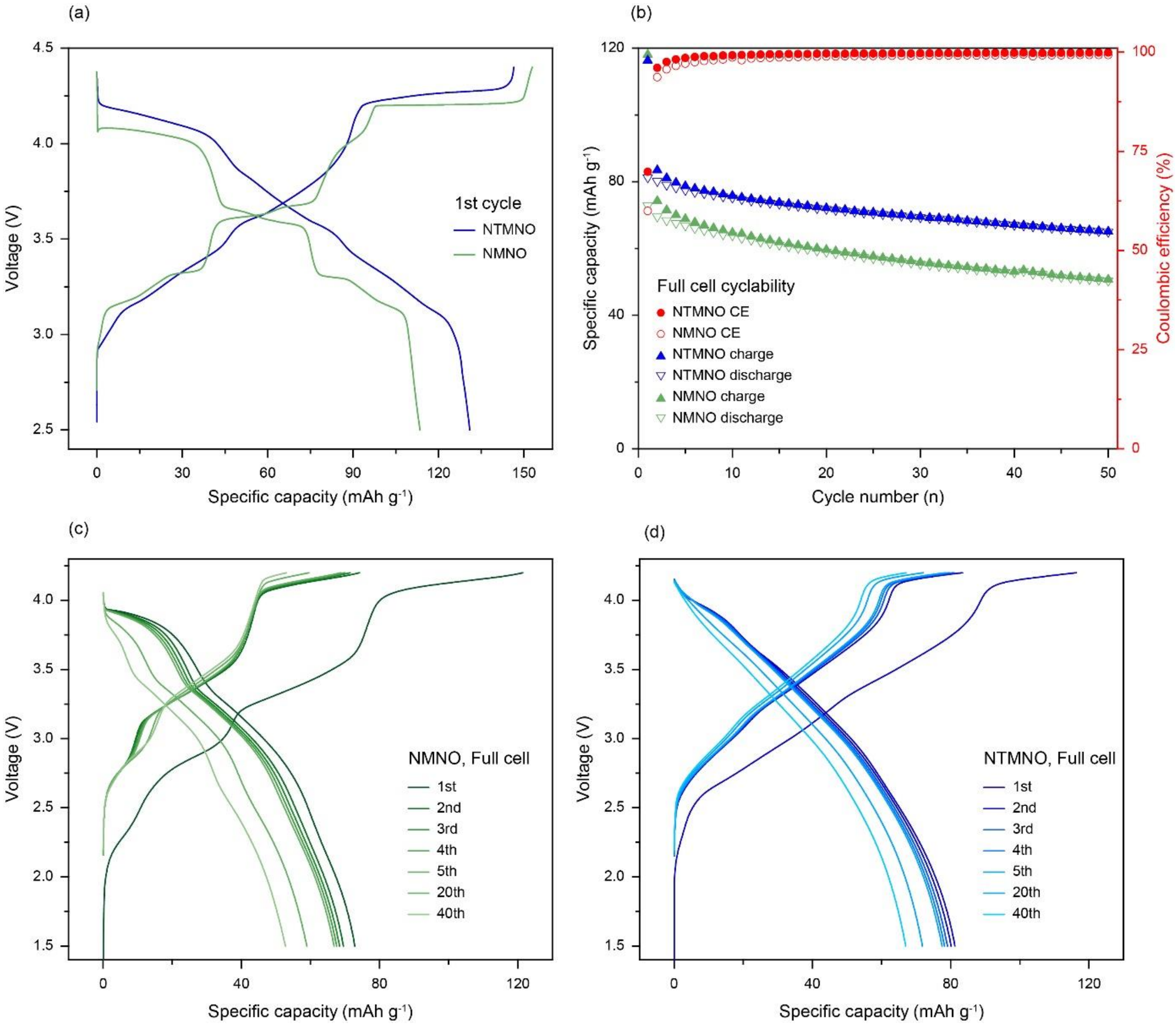

Figure S6. Electrochemical properties with half-cell and full-cell conditions of NMNO and NTMNO. (a)Voltage plot of NMNO and NTMNO at the current density of 30 mA g^{-1} with half-cell condition. (b) Full-cell cyclability data of both cathodes at the current density of 300 mA g^{-1} and corresponding voltage plot of (c) NMNO and (d) NTMNO.
